# Solubility analysis of homologous series of amino acids and solvation energetics in aqueous potassium sulfate solution

**DOI:** 10.1016/j.heliyon.2019.e02304

**Published:** 2019-08-23

**Authors:** Aslam Hossain, Kalachand Mahali, Bijoy Krishna Dolui, Partha Sarathi Guin, Sanjay Roy

**Affiliations:** a Department of Physical and Inorganic Chemistry, Institute of Natural Sciences and Mathematics, Ural Federal University, 620000, Yekaterinburg, Russia; b Department of Chemistry, University of Kalyani, Nadia, 741235, West Bengal, India; c Department of Chemistry, Visva-Bharati, Santiniketan, Birbhum, 731235, West Bengal, India; d Department of Chemistry, Shibpur Dinobundhoo Institution (college), Howrah, 711102, West Bengal, India

**Keywords:** Physical chemistry, Theoretical chemistry, Electrolyte solution, Salting-out effect, Transfer gibbs free energies, Salting-in effect

## Abstract

In this study we estimated the solubilities of glycine, D,L-alanine, D,L-nor-valine and D,L-serine in aqueous mixtures of potassium sulfate (K_2_SO_4_) at 298.15 K using analytical ‘gravimetric method’. The experimental solubilities of homologous series of amino acids in aqueous K_2_SO_4_ mixture were discussed in terms of relative solubility, salting-in and salting-out effect by evaluating the influential constants. The effect of physicochemical and chemical factors on solubility were discussed briefly and correlated with the thermodynamics. Initially, the study of solvation energetics such as transfer Gibbs energies were evaluated based on the calculations from solubility data and relative stability of the experimental molecules was discussed under the experimental condition.

## 1 Introduction

For quite a long time a significant attention has been made to study the thermodynamics of biologically important small molecules such as amino acids in dilute aqueous electrolyte solutions. It provides valuable information about the nature of the interactions between polar and nonpolar groups, water and aqueous electrolytes and thus contributes in understanding the chemistry of protein like complex systems in aqueous medium [1, 2, 3, 4, 5, 6]. In this case the solubility data are necessary to explain the thermodynamics and useful in the clarification of solute–solute and solute–solvent interactions [7, 8]. Solubility is also important to design and optimize various industrial processes such as chemical, pharmaceutical, food, cosmetics and biodegradable plastic industries [9, 10, 11, 12]. The solvation thermodynamics of amino acids also play crucial role in dissolution and purification of proteins [13].

Glycine is the simplest amino acid having no hydrophobic side chain whereas D,L-alanine and D,L- nor-valine consist of hydrophobic side chains such as CH_3_- and CH_3_–CH(CH_3_)-, respectively. On the other hand, D,L-serine contains a hydrophobic aliphatic hydrocarbon group (–CH_2_-) attached with one hydrophilic hydroxyl (–OH) moiety [Table 1]. These structural differences may affect solvation factors which are very important for their separation from excess reagents and other impurities in aqueous solution. This is a demanding task which is often done by crystallization or precipitation processes [14]. Interestingly the separation price of amino acids has been found as about 50% of the entire production cost [7, 11, 14]. The influence of electrolytes ions has a significant role on separation of amino acids from raw materials. So, the solubility study of amino acids in the presence different electrolytes helps us to draw an idea in designing appropriate model for the purification of different amino acids.

**Table 1:** Specification of chemical samples.

Name	Source	Initial purity^a^ (% of mass fraction purity)	Purification method
Glycine	E. Merck, INDIA	99.8 % (mass)	Dried in vacuum desiccator
DL-alanine	E. Merck, INDIA	99.8 % (mass)	drying in a dehydrator with silica gel
DL-nor-valine	E. Merck, INDIA	99.8 % (mass)	Dried in vacuum desiccator
D,L-serine	E. Merck, INDIA	99.8 % (mass)	drying in a dehydrator with silica gel
Potassium sulfate	E. Merck, INDIA	99 %	Oven dried
Water			distillation

a Stated by the supplier.

The aim of the present work is to find out the effect of hydrophobic alkyl group and the influence of ionic –NH_2_ and –COOH groups on the solubility of the experimental amino acids in the presence of electrolyte. On the other hand the amino acids have been used extensively as model compound for more complex biomolecules such as proteins, but a more thoughtful understanding of the electrolyte effect on amino acid solutions is still desirable to reveal the molecular interactions between salts and protein functional groups [1, 2, 3, 4, 5, 6]. From the theoretical point of view the interactions between electrolyte ions and small molecules with biological macromolecules are of substantial importance in determining the nature of macromolecules. In particular, information on the thermodynamic solvation properties of amino acids in aqueous salt solutions helps to realize the conformational changes of molecules in solution produced by the addition of denaturants, or by the transport of charged solutes across membranes. So, we choose homologous series of amino acids such as glycine, D,L-alanine, D,L-nor-valine and D,L-serine and estimated saturated solubility in the presence of aqueous K_2_SO_4_ solution at 298.15 K using gravimetric method [15, 16, 17, 18, 19]. We introduced our attention to find out the physicochemical and chemical factors associated with the solubility and the transfer Gibbs free energies and explained the relationship among solubility, solvation thermodynamics and stability of the studied amino acids in aqueous K_2_SO_4_ solution at 298.15 K. The research will definitely be helpful to improve our knowledge in the field of amino acid research such as chemical, physical, biochemical, engineering, pharmaceutical and industrial sciences.

## 2 Experimental

### 2.1 Chemicals and purifications

Glycine (E. Merck, INDIA), D,L-alanine (E. Merck, INDIA), D,L-nor-valine (>99.8%, Sigma Aldrich) and D,L-serine (>99.8 %, E. Merck, INDIA) were used after drying in vacuum desiccators at 370 K for 7 days*.* Potassium sulfate (K_2_SO_4_) of purity 99 % procured from E Merck, India. It was then dried in hot air oven at 500 K for 7 days and kept it for 3 days in vacuum desiccator prior to use. Triple distilled water (conductivity 0.6 μS/cm) was used in the entire study to prepare all aqueous solutions. Specifications of the compounds were summarized in Table 1.

### 2.2 Preparations of saturated solutions and solubility measurement

The aqueous solution of K_2_SO_4_ with the concentrations of 0.0, 0.20, 0.30, 0.40, 0.50 and 0.65 in molality were prepared by dissolving required amount of K_2_SO_4_ in triple distilled water.

A low-to-high temperature controlling thermostat with an accuracy of ±0.10 K at atmospheric pressure (p = 0.1 MPa) was used for all measurements. The first step of the gravimetric method [15, 16, 17, 18, 19] requires the preparation of a saturated solution of amino acids in a particular electrolyte solution having a certain concentration of K_2_SO_4_ at studied temperature and this solution was taken in a jacketed glass cell. The temperature was controlled at 298.15K by circulating thermo stated water in the jacket and such studied solution was continuously stirred for 12 h to achieve saturation equilibrium. The mixing process was then stopped and kept for 7h to settle down the undissolved amino acid. 5 mL of such saturated solution was collected from the clear phase using a dried pipette within a few seconds. The collected saturated solution was then filtered by using a 0.22 μm HPLC disposable filter and kept instantly into glass vessels and weighted. The solution was heated and evaporated to obtain dried mass in hot air over at 350 K. The dried mass was then cooled in a dehydrator containing silica gel for 24 h and weighed. The process was repeated till a constant mass appeared. For each experimental amino acid the above mentioned process was repeated thrice at desired temperature for a particular composition of the electrolyte and average value of the solubility of amino acid was determined. The solubility values in three measurements were found to be agreed within 2.5 %.

## 3 Results and discussion

### 3.1 Solubility and salting-in/salting-out effects

The mass of the dissolved amino acid in each 5 ml solution can be measured by knowing the amount of electrolyte in such solution (W_1_ g), weight of the empty glass vessel (W_2_ g), and glass vessel with dry sample (W_3_ g). If, the concentration of the electrolyte is ‘c’ then weight of 5 mL electrolyte salt would be W_1_ = (Mc×5/1000) g, where M is the molar mass of the electrolyte. Thus the weight of amino acid would be W = (W_3_─ W_2_─ W_1_) [20, 21]. Such weight of amino acid was converted to solubilities in mole per kg of pure water in the absence and presence of electrolyte (K_2_SO_4_). Though it is to be important to note that in many previous studies [13, 21, 22] it has shown that that no weighable amount of precipitation or adsorption of electrolyte (K_2_SO_4_ in this study) on amino acid in solid phase will occur even in different content of amino acid as well as electrolyte mixtures. That is why in this study we also perform atomic absorption spectroscopy was done to make sure the chance of adsorption or assimilation of the salt and degradation of the sample on the solid-phase of the amino acids, in the mixture as it was done in previous works [13, 21, 22]. Concentrations of cations in the aqueous electrolyte and in the amino acid–water-electrolyte systems were also compared to validate the fact that the electrolytes were not absorbed or incorporated on the solid phase of the amino acids i.e., the precipitate was formed only by the amino acid [13, 21, 22]. Electrolyte solutions containing different concentration of amino acids in excess to saturation were made and cation concentrations were measured for comparison of cation concentration in each solution. The highest difference in the experimental results was observed as ±0.005 mol kg^−1^. This means that, in-spite of the existence of different amounts of amino acid in the solution, no significant quantity of electrolyte was precipitated or adsorbed on the solid phase of amino acid [13, 21, 22]. This must proves that solid recovered was only the amino acids [21, 22].

**Fig. 1:**
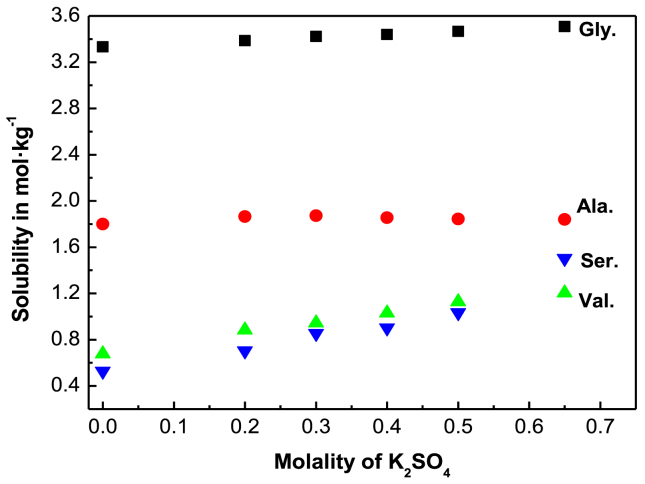
Variation of solubilities (mol·kg^−1^) with molality of K_2_SO_4_ in aqueous K_2_SO_4_ solution for glycine (■), D,L-alanine (●),D,L-serine (▼) and D,L-nor-valine (▲) at 298.15 K.

**Fig. 2:**
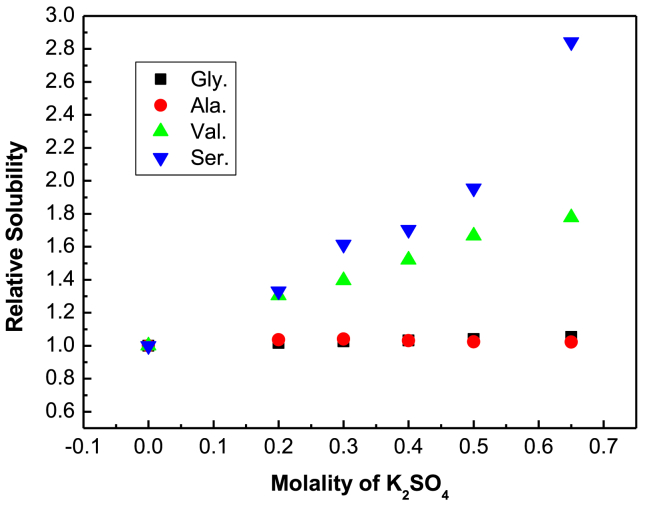
Relative solubility of glycine (■), D,L-alanine (●),D,L-serine (▼) and D,L-nor-valine (▲) with the molality of K_2_SO_4_ in aqueous K_2_SO_4_ solution at 298.15 K.

Solubilities of the amino acids in aqueous medium in the absence and presence of K_2_SO_4_ salt in different concentrations are summarized in Table 2 and variation is shown in Fig. 1. Results show that all the experimental amino acids are more soluble in the presence of K_2_SO_4_ whereas only D,L-alanine is less soluble after 0.30 molal concentration of K_2_SO_4_ under all experimental conditions. The relative solubilities also show the related changes in solubilities shown in Table 3 & in Fig. 2. We also presented (Table 2) the literature solubility data done by Farid I. El-Dossoki [21] for the same amino acids. In this regard it is to be said that the highest solubility of K_2_SO_4_ is 0.689 mol/kg in water at 298.15 K. But EI-Dossoki used 1.0 mol/kg K_2_SO_4_ solution at same temperature and measured the solubility upto that electrolyte concentration. But we are unable to make 1.0 mol/kg K_2_SO_4_ aqueous solution at 298.15 K. Definitely there was some error in the EI-Dossoki [21] result in higher electrolyte concentration. Though the solubility trend is same however the literature results [21] show slight higher solubility for all the experimental amino acids in aqueous electrolyte K_2_SO_4_ solution only exceptions are found in pure water for D,L– nor-valine and D,L-serine. In pure water, D,L –nor-valine and D,L-serine show more solubility in the present work than the literature data. These differences might be due to use of different experimental set up, chemical used in the investigation in the earlier studied by Farid I. El-Dossoki [21].

**Table 2:** Solubility (S) of glycine, D,L-alanine, D,L-nor-valine and D,L-serine in pure water and aqueous K_2_SO_4_ solution in mol·kg^−1^ at 298.15 K and atmospheric pressure (p = 0.1 MPa^**a**^) in the present study and in the literature [21].

Molality of salt (m)	Solubility (S) in mol·kg^−1^ at 298.15 K			
	Glycine	D,L-alanine	D,L-nor-valine	D,L-serine
0.00	3.332	1.800	0.677	0.529
	3.338 [21]	1.895 [21]	0.605 [21]	[0.479]
0.20	3.386	1.864	0.883	0.704
0.30	3.422	1.872	0.945	0.854
0.40	3.440	1.856	1.030	0.902
0.50	3.466	1.844	1.128	1.035
	4.195 [21]	2.489 [21]	1.149 [21]	1.023 [21]
0.65	3.508	1.839	1.204	1.504

u(T) = ±0.10 K; u(m) = ±0.01; relative uncertainties of pressure is u_r_(p) = 0.02^a^.

**Table 3:** Relative solubility (S_s_/S_R_) and log (S_S_/S_R_) of glycine, D,L-alanine, D,L-nor-valine and D,L-serine in aqueous K_2_SO_4_ solution in different compositions of K_2_SO_4_ at 298.15 K and atmospheric pressure (p = 0.1 MPa^a^) in the present study.

Molality of KNO_3_(m)	Relative Solubility (S_s_/S_R_) at 298.15 K	log (S_S_/S_R_)_298.15K_
Glycine		
0.20	1.016	0.00698
0.30	1.027	0.01158
0.40	1.032	0.01385
0.50	1.040	0.01712
0.65	1.052	0.02235
D,L-alanine		
0.20	1.036	0.01517
0.30	1.040	0.01703
0.40	1.031	0.01331
0.50	1.024	0.01049
0.65	1.022	0.00931
D,L-nor-valine		
0.20	1.304	0.11537
0.30	1.396	0.14484
0.40	1.521	0.18225
0.50	1.666	0.22172
0.65	1.778	0.25004
D,L-serine		
0.20	1.331	0.12411
0.30	1.614	0.20800
0.40	1.705	0.23175
0.50	1.956	0.29148
0.65	2.843	0.45379

u(T) = ±0.10 K; u(m) = ±0.01; relative uncertainties of pressure is u_r_(p) = 0.02^a^.

**Fig. 3:**
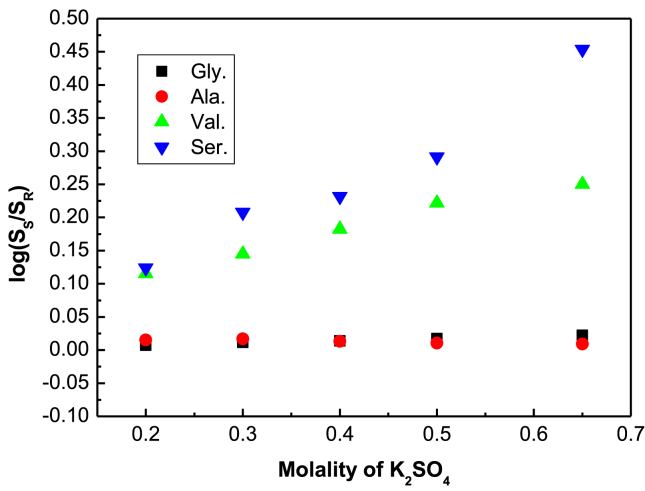
Logarithm of the ratio of solubilities S_S_ with and S_R_ without electrolytes for glycine (■), D,L-alanine (●),D,L-serine (▼) and D,L-nor-valine (▲) with the molality of K_2_SO_4_ in aqueous K_2_SO_4_ solution at 298.15 K.

It clears from the solubility data that the electrolyte influences the solubilities of the amino acids notably [18, 23, 24, 25, 26]. In previous studies by various researchers [27, 28, 29, 30] it was shown that the solubilities of the amino acids were affected greatly by the temperature of the solvation media. The regular increment of solubility glycine, D,L-nor-valine and D,L-serine in the presence of increasing concentration of K_2_SO_4_ probably is due to the ‘salting-in effect’ [21, 30]. This effect mainly comes up due to the interactions of the electrolyte ions (K^+^ and SO_4_^2-^) and water molecules with the hydrocarbon backbone and charged amino and carboxyl groups of zwitterionic amino acids. It is to be noted that crystal energy is a major factor for the solubility of the amino acids. Between glycine and DL-alanine, DL-alanine has higher crystal energy thus it shows lower solubility in the experimental solutions. Although the solubility of DL-alanine shows a slight increment in presence of electrolyte K_2_SO_4_ upto 0.30 molality but after that the solubility decreases. In lower concentration of electrolyte DL-alanine forms ion-pair complexes strongly with the cation and anion of the electrolyte due to good agreement of sizes of zwitterion and electrolyte ions. The ion pair complex is a complex which is formed in between zwitterion of the amino acid and the electrolyte anion and cation. The amino acids, existing as zwitterions A^−^A^+^ in the solution system, may form soluble ion pair complexes (due to cavity forming interaction) like A^−^A^+^ + C^+^X^−^ ↔ C^+^(A^−^A^+^) X^−^ with the cation C^+^ (here K^+^) and anion X^−^ (here SO_4_^-^) of such electrolyte. The difference in the solubility trends of amino acids in the absence and presence of electrolytes which is observed as salting-in or salting-out effect is most likely due to this kind of complexes formed in the aqueous solution by the different amino acids with the cations and anions. In lower concentration the ion-pair formation gets most favourable which suggests salting-in effect. On the other hand in higher concentration of electrolytes the ion-pair formation is diminished because there might be grow the steric hindrance and cationophilic interaction towards the ion-pair complexes hence solubility of DL-alanine is decreased according to its crystal energy. This type of solubility effect for D,L-alanine also found in many previous literature in different experimental conditions [17, 18, 31, 32]. Mainly the chemical structures and structural orientation of the amino acid comprises a crucial depending ability for the ‘salting-in and ‘salting-out’ effects [21, 30]. To realize the ‘salting-in and ‘salting-out’ effects very precisely the relative solubility measurement for the amino acids at a particular temperature is very imperative at each point of electrolyte concentrations. The trend of salting-out and salting-in effects are given and explain by the constant, *K*_*si*_, the quantitative estimate of salting-in and salting-out effects which are determined by the use of Eq. (1) [7, 19, 21] and offered in Table 4.

(1) $$\log\left(\frac{S_{S}}{S_{R}}\right)=\;K_{si}C$$

where, S_S_ is the solubility of amino acid in aqueous K_2_SO_4_ mixtures with concentration (C) in molality, and S_R_ is the solubility of respective amino acid in pure water. The log (S_S_/S_R_) values are used from Table 3 and log (S_S_/S_R_) *vs.* ‘C’ in molality plot is shown by Fig. 3. The linear relationship of log (S_S_/S_R_) *vs*. ‘C’ was then employed to estimate the values of *K*_*si*_ for the experimental amino acids which are shown in Table 4. The observed values of *K*_*si*_ in aqueous K_2_SO_4_ solvent system provide necessary proofs to the trend in present experimental solubility results stated in Table 2. The finding positive values of *K*_si_ support the experimental conclusions on salting-in effect of K_2_SO_4_ for glycine, D,L-nor-valine and for D,L-serine and the slight negative value of the constant indicates salting-out effect for D,L-alanine in aqueous K_2_SO_4_ solvent system (Table 4).

**Table 4:** Salting-in and Salting-out constants of glycine, D,L-alanine, D,L-nor-valine and D,L-serine in presence of K_2_SO_4_ in solution at 298.15 K

Amino Acids	*K*_*si298.15 K*_
Glycine	0.03301 ± 0.00166
D,L-alanine	− 0.0165 ± 0.00459
D,L-nor-valine	0.31133 ± 0.02334
D,L-serine	0.68756 ± 0.08783

The more positive value of *K*_si_ indicates more salting-in effect and negative value indicate salting-out effect. For the present amino acids the trend of salting-in effect is as follows: D,L-alanine < glycine < D,L-nor-valine < D,L-serine (Table 4).

### 3.2 Transfer free energetics

The molal solubilities in the aqueous-electrolyte as well as in pure water were used to determine apparent standard Gibbs energy of solutions ($\Delta G_{s}^{0}\left(i\right)$) on molal scale using Eq. (2) [33, 34, 35, 36].

(2) $$\Delta G_{S}^{0}\left(i\right)\approx \;-RT\;\text{ln}\left(S\right)$$

‘S’ is the experimental saturated solubility of the amino acids in mol·kg^−1^. The apparent standard Gibbs energy of solutions ($\Delta G_{s}^{0}\left(i\right)$) were shown in Table 5.

**Table 5:** Values of ΔG_s_^0^(i) from present experimental solubility and literature solubility of glycine, D,L-alanine, D,L-nor-valine and D,L-serine in aqueous K_2_SO_4_ solution in kJ·mol^−1^.

Molality of salt	ΔG_s_^0^(i) from present solubility kJ·mol^−1^			
	Glycine	D,L-alanine	D,L-nor-valine	D,L-serine
0.00	−2.983	−1.457	0.967	1.578
0.20	−3.023	−1.544	0.308	0.870
0.30	−3.049	−1.554	0.140	0.391
0.40	−3.063	−1.533	−0.073	0.256
0.50	−3.081	−1.517	−0.299	−0.085
0.65	−3.111	−1.510	−0.460	−1.012

**Fig. 4:**
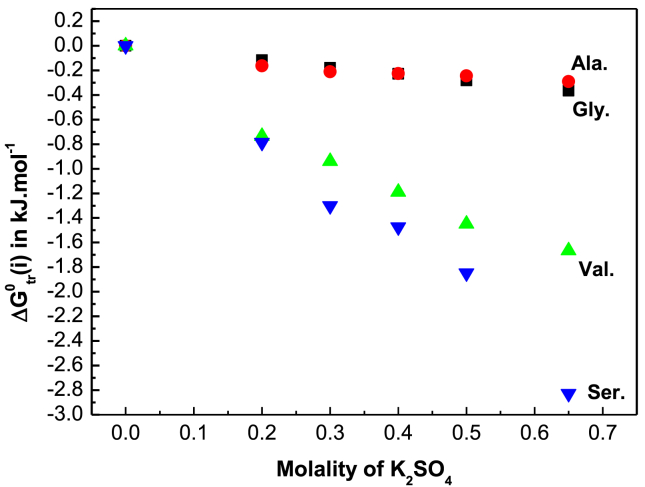
Variation of $\Delta G_{tr}^{0}$(i)in kJ·mol^−1^with molality of K_2_SO_4_ in aqueous K_2_SO_4_ solution for glycine (■), D,L-alanine (●),D,L-serine (▼) and D,L-nor-valine (▲) at 298.15 K.

The transfer Gibbs energy of solutions was calculated by Eq. (3) [37, 38].

(3) $$\Delta G_{tr}^{0}\left(S\right)=\;RT\;\ln\left(S_{R}/S_{s}\right)$$

where, the subscripts R and S are for water and aqueous-electrolyte respectively. The standard transfer free energies in mole fraction scale, $\Delta G_{tr}^{0}\left(i\right)$was calculated by [11, 38].

(4) $$\Delta G_{tr}^{0}\left(i\right)=\Delta G_{tr}^{0}\left(s\right)-RT\;\ln\left(M_{s}/M_{R}\right)$$

where M_s_ and M_R_ refer to the molar mass of electrolyte (K_2_SO_4_) mixture and reference solvent (water) respectively. The values of $\Delta G_{tr}^{0}\left(i\right)$ are shown in Table 6. Fig. 4 corresponds to the variation of $\Delta G_{tr}^{0}\left(i\right)$ for the amino acids with molality of K_2_SO_4_ at 298.15.

**Table 6:** Gibbs energies of transfer $\Delta G_{tr}^{0}$(i), $\Delta G_{tr,cav}^{0}$(i), $\Delta G_{tr,dd}^{0}$(i), $\Delta G_{tr,ch}^{0}$(i) of glycine, D,L-alanine, D,L-nor-valine and D,L-serine in aqueous K_2_SO_4_ solution in kJ·mol^−1^.

Molality of KNO_3_ (m)	$\Delta G_{tr}^{0}$(i) kJ mol^−1^	$\Delta G_{tr,cav}^{0}$(i) kJ mol^−1^	$\Delta G_{tr,dd}^{0}$(i) kJ·mol^−1^	$\Delta G_{tr,ch}^{0}$(i) kJ mol^−1^
Glycine				
0	0	0	0	0
0.20	−0.116	−0.230	0.013	0.101
0.30	−0.179	−0.340	0.035	0.126
0.40	−0.229	−0.448	0.059	0.160
0.50	−0.282	−0.546	0.086	0.178
0.65	−0.365	−0.697	0.149	0.183
D,L-alanine				
0	0	0	0	0
0.20	−0.163	−0.247	0.011	0.073
0.30	−0.211	−0.365	0.029	0.125
0.40	−0.226	−0.480	0.049	0.205
0.50	−0.244	−0.585	0.073	0.268
0.65	−0.291	−0.747	0.127	0.329
D,L-nor-valine				
0	0	0	0	0
0.20	−0.735	−0.274	0.009	−0.470
0.30	−0.940	−0.405	0.023	−0.558
0.40	−1.190	−0.531	0.039	−0.698
0.50	−1.449	−0.648	0.058	−0.859
0.65	−1.665	−0.826	0.099	−0.938
D,L-serine				
0	0	0	0	0
0.20	−0.785	−0.240	0.006	−0.551
0.30	−1.300	−0.354	0.016	−0.962
0.40	−1.473	−0.465	0.026	−1.034
0.50	−1.848	−0.568	0.039	−1.319
0.65	−2.828	−0.724	0.067	−2.171

The required diameter of glycine, DL-alanine, DL-nor-valine and DL-serine are 5.64 Å, 6.16 Å, 6.92 Å and 5.93 Å respectively [15, 16, 27]. The dipole moment of glycine, DL-alanine, DL-nor-valine and DL-serine are 15.7 D, 15.9 D, 16.0 D and 11.10 D respectively [15, 16, 27].

The $\Delta G_{tr}^{0}\left(i\right)$may be ascribed as the sum of the following terms neglecting the contribution of dipole-induced dipole term [15, 16, 27, 28, 34]:

(5) $$\Delta G_{tr}^{0}\left(i\right)=\Delta G_{tr,cav}^{0}\left(i\right)+\Delta G_{tr,d-d}^{0}\left(i\right)+\Delta G_{tr,ch}^{0}\left(i\right)$$

Here, $\Delta G_{tr,cav}^{0}\left(i\right)$ represents the transfer free energy due to cavity effect of species in pure water and aquo-ionic media. $\Delta G_{tr,d-d}^{0}\left(i\right)$ is due to dipole-dipole interaction between dipolar amino acid and solvent molecules. $\Delta G_{tr,ch}^{0}\left(i\right)$signifies the effects rising from acid-base or short-range dispersion interaction, hydrophilic or hydrophobic hydration and structural effects. $\Delta G_{tr,cav}^{0}\left(i\right)$were calculated based on scaled particle theory [27, 39, 40]. According to this theory, we can assume that solute and solvent molecules are equivalent hard sphere models as dictated by their respective diameters (Table 7a and b). Eq. (6) was applied in calculating cavity [27, 28, 34, 39] as follows:

(6) $$\Delta G_{cav}^{0}\left(i\right)=G_{C}+RT\;\ln\left(RT/V_{S}\right)$$

Where

**Table 7a:** Values of solute/solvent parameters: mole fraction of K_2_SO_4_ (Z_s_), molality of K_2_SO_4_ in water (m), mole fraction of water (Z_R_), molar mass of cosolvent (M_s_), density (d_s_), hard sphere diameter of co-solvent ($\sigma_{x}$) (K_2_SO_4_+H_2_O) and apparent dipole moment of co-solvent ($\mu_{s}$), and thermal expansibility constant (α) of H_2_O + K_2_SO_4_ solution at 298.15K.

Molality of K_2_SO_4_ (m)	Mole fraction (z_s)_	Mole fraction (z_R_)	Molar mass (M_S_)	Density (ds) (kg. dm-^3^)	Molar Vol.(Vs) (dm^3^.mol^−1^)	$\sigma_{s}$ (nm)	Apparent Dipole Moment ($\mu_{s}$) (D)	α (x 10^3^)
0.00	0.0000	1.0000	18.015	0.9970^#^	18.06921	0.274	1.831*	0.257*
0.20	0.0036	0.9964	18.5775	1.00299	18.52212	0.275	1.831	0.257
0.30	0.0054	0.9946	18.8587	1.00598	18.7466	0.276	1.831	0.257
0.40	0.0072	0.9928	19.1399	1.00897	18.96974	0.276	1.831	0.257
0.50	0.0089	0.9911	19.4056	1.0118	19.17928	0.276	1.831	0.257
0.65	0.0116	0.9884	19.8274	1.01629	19.50959	0.277	1.831	0.257

u(T) = ± 0.10 K; ^#,^ * For reference [40].Density, molar mass, size and dipole moment values of K_2_SO_4_, 2.66 g mol^−1^, 174.259, and 5.92 Å respectively are taken from reference [19] and internet sources.The required diameter of glycine, DL-alanine, DL-nor-valine and DL-serine are 5.64 Å, 6.16 Å, 6.92 Å and 5.93 Å respectively [15, 16, 27]. The dipole moment of glycine, DL-alanine, DL-nor-valine and DL-serine are 15.7 D, 15.9 D, 16.0 D and 11.10 D respectively [15, 16, 27].

**Table 7b:** Values of $\sigma_{s-x}=\frac{1}{2}\left(\sigma_{s}+\sigma_{x}\right)$ of the amino acids present in water-electrolytes systems at 298.15 K.

Molality of K_2_SO_4_ (m)	$\sigma_{s}$ (nm)	$\sigma_{s-x}$ (nm) Glycine	$\sigma_{s-x}$ (nm) D,L-alanine	$\sigma_{s-x}$ (nm) D,L- nor-valine	$\sigma_{s-x}$ (nm) D,L-serine
0.00	0.274	0.419	0.445	0.483	0.434
0.20	0.275	0.419	0.445	0.483	0.434
0.30	0.276	0.420	0.446	0.484	0.435
0.40	0.276	0.420	0.446	0.484	0.435
0.50	0.276	0.420	0.446	0.484	0.435
0.65	0.277	0.421	0.447	0.485	0.436

The required diameter of glycine, DL-alanine, DL-nor-valine and DL-serine are 5.64 Å, 6.16 Å, 6.92 Å, 5.93 Å and water 2.74 Å respectively were taken from refs. [15, 16, 27, 40].

${G_{C}=RT[-\ln(1-Z)+\{3X/(1-Z)\}\sigma_{x}+\{3Y/(1-Z)\}{\sigma_{x}}^{2}+\{9X^{2}/2(1-Z)}^{2}\}{\sigma_{x}}^{2}]$$Ζ=\pi N_{A}/6V_{s}\left(z_{R}{\sigma_{R}}^{3}+z_{s}{\sigma_{s}}^{3}\right)$$X=\pi N_{A}/6V_{s}\left(z_{R}{\sigma_{R}}^{2}+z_{s}{\sigma_{s}}^{2}\right)$$Y=\pi N_{A}/6V_{s}\left(z_{R}\sigma_{R}+z_{s}\sigma_{s}\right)$$V_{s}={Μ}_{s}/d_{s}$

where *N*_*A*_ is the Avogadro's number, z_R_ and z_s_ are the mole fraction of water and salts respectively. ‘$\sigma_{x}$’, ‘$\sigma_{R}$_’_and ‘$\sigma_{s}$’ stand for hard sphere diameters of amino acids, water and co-solvent respectively. M_s_ is the molar mass of the electrolyte solvents whereas ‘d_s_’ is molar density of the same.

Finally, $\Delta G_{tr,cav}^{0}\left(i\right)$represents the difference.

(7) $$\Delta G_{tr,cav}^{0}(i){=\text{ ​}}_{S}\Delta G_{cav}^{0}(cav){-}_{R}\Delta G_{cav}^{0}(cav)=(SG_{c}{-}_{R}G_{c})+RT\;\ln(V_{R}/V_{s})$$

Appropriate solvent parameters of Table 7a and b were used to calculate $\Delta G_{tr,cav}^{0}\left(i\right)$ .

The $\Delta G_{tr,d-d}^{0}\left(i\right)$ values were evaluated as shown below [27, 34].

(8) ΔGtr,d−d0(i)=(ΔSGd−d0(i)−RΔGd−d0(i))

In a solvent, ‘s’, the expression of ΔsGd−d0(i)is described as follows:

ΔsGd−d0(i)=−(8Π/9)N2μs2μx2σs−x−3(kT)−1Vx−1=A/TVs;

$${\text{where ​}A=-\left(8\Pi /9\right)N^{2}\mu_{s}^{2}\mu_{x}^{2}\sigma_{s-x}^{-3}\left(k\right)}^{-1}$$

(9) $$\text{and ​}\;V_{s}=M_{s}/d_{s}$$

Here N is the Avogadro^'^s number whereas $\mu_{s}$ and $\mu_{x}$are the dipole moments of solvent and amino acid molecules, respectively (Table 7a and b), $\sigma_{s-x}$ expresses the distance in which the attractive and repulsive interactions between the solvent and solute molecules are the same and it is generally equal to $\frac{1}{2}\sigma_{s}+\sigma_{x}$, where $\sigma_{s}$ and $\sigma_{x}$are the hard sphere diameter of solvent and solute molecules, respectively. Here $\mu_{x}$and $\sigma_{s}$for such mixed binary solvent system are computed with the variation of mole fraction and are summarized in Table 7a and b. The quantity was further multiplied by the term $X_{s1}$ following of Marcus [40] in order to obtain $\Delta G_{tr,d-d}^{0}\left(i\right)$term on mole fraction scale. The expression of $X_{s1}$ is given as:

(10) $$X_{s1}=X_{s}\left(\mu_{s}/\sigma_{s}^{3}\right)/\left(\mu_{R}/\sigma_{R}^{3}\right)$$

It is important to note that $X_{s1}$ is the real mole fraction contribution owing to the dipole-dipole interaction.

The values of $\Delta G_{tr}^{0}\left(i\right)$ (Fig. 4) show a negative trend for all the experimental amino acids except D,L-alanine which shows a slight deviation in higher concentration of K_2_SO_4_ in solution. The $\Delta G_{tr}^{0}\left(i\right)$ results suggest that D,L-alanine is slightly unstable in higher mass of K_2_SO_4_ in solution whereas glycine, D,L-nor-valine and D,L-serine are more stable in the aqueous potassium sulfate solution rather than in pure water.

The $\Delta G_{tr,cav}^{0}\left(i\right)$values (Table 6) for the amino acids show that DL-nor-valine is more stable whereas glycine shows less stable in aqueous mixtures of K_2_SO_4_. The order of stability is as: glycine < DL-serine < DL-alanine < DL-nor-valine. The observed stability order explained that the comparatively larger amino acids forms cavity easily in water-electrolyte (K_2_SO_4_) mixture rather than in pure water. It is because $\Delta G_{t,cav}^{0}\left(i\right)$ values are directed by the hard sphere diameter of solute, solvent and density of the solvent mixtures (Eq. 6). The hard sphere diameter of K_2_SO_4_ (5.92 Å) [19] is higher than that of pure water molecule (2.74 Å) [30, 34, 40] and therefore the solvated K_2_SO_4_ mixture is more suitable for cavity creation for the larger amino acid than the smaller amino acid.

Δ$G_{t,d-d}^{0}$(i) values show negative increment in the order: glycine < D,L-alanine < D,L-nor-valine < D,L-serine, in aqueous electrolytes solvent systems (Table 6). This directs to conclude that D,L-serine is more stabilized by the dipole-dipole interaction between the solute and solvent molecules in both the aqueous electrolyte solutions.

Here Δ$G_{t,d-d}^{0}$(i) values, which are acquired after subtraction of _R_Δ$G_{t,d-d}^{0}$(i) from _S_Δ$G_{t,d-d}^{0}$(i), depend on dipole moment of solute and co-solvents system and on the hard-sphere diameter of solute and co-solvent molecules.

Since, Δ$G_{tr}^{0}$(i) = Δ$G_{tr,cav}^{0}$(i) + Δ$G_{tr,d-d}^{0}$(i) +Δ$G_{tr,ch}^{0}$(i)

**Fig. 5:**
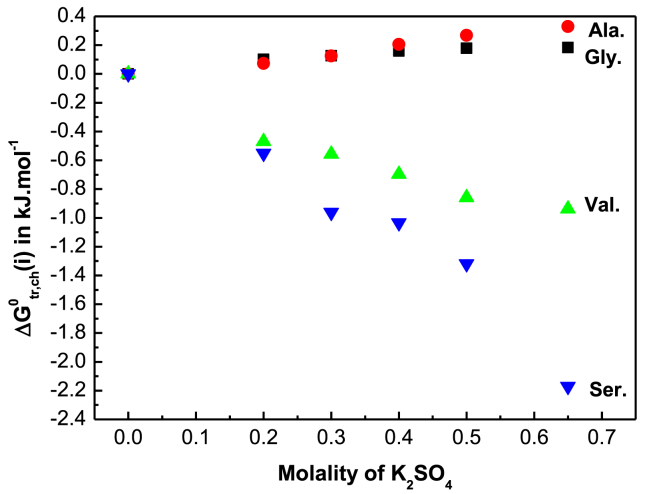
Variation of $\Delta G_{tr,ch}^{0}$(i)in kJ·mol^−1^with molality concentration of K_2_SO_4_ in aqueous K_2_SO_4_ solution for glycine (■), D,L-alanine (●),D,L-serine (▼) and D,L-nor-valine (▲) at 298.15 K.

Hence, the values $\Delta G_{tr,cav}^{0}\left(i\right)$and $\Delta G_{tr,d-d}^{0}\left(i\right)$are subtracted from $\Delta G_{tr}^{0}\left(i\right)$to get $\Delta G_{tr,ch}^{0}\left(i\right)$ of amino acid and the values are shown in Table 6. The value of $\Delta G_{tr,ch}^{0}\left(i\right)$is illustrated in Fig. 5.

In the solute solvent mixed system, there involves various types of chemical and other interactions such as partial Gibbs energy of activation of viscous flow per mole of solvent, and per mole of solute, viscosity, acid-base, H-bonding, hydrophilic and hydrophobic interactions, hard-soft and dispersion interactions depending upon the structural variations of the solute and solvent molecules [41, 42, 43, 44, 45, 46].

From Fig. 5 it is observed that D,L-serine and D,L-nor-valine shows more stability rather than glycine and D,L-alanine in higher concentration of electrolyte solution. Among the four amino acids, DL-serine shows the highest stability in the electrolytes solvent systems. Hence the chemical stability order of these amino acids in aqueous K_2_SO_4_ is as follow: D,L-serine > D,L- nor-valine > glycine > D,L-alanine.

The observed stability order of the amino acids having structural differences arises mainly due to the presence different side chains in their structure (Table 1). D,L-serine contains –OH group whereas other three amino acids do not contain OH group. The nonexistence of –OH group in other three amino acids show lesser dipole-dipole, hydrophilic and acid-base typesof chemical interactions with aqueous as well as aqueous K_2_SO_4_ solutions. Butthe former types of interactions are strongly favourable for DL-serine which directs to its maximum stability among the present four amino acids in the aqueous electrolytesolvent systems.

D,L-nor-valine is the second most stable in aqueous K_2_SO_4_ solutions in terms of chemical types of interaction because summation of negative trends of cavity forming and dipole-dipole interactions overcomes the total transfer free energetics. This leads to negative chemical transfer Gibbs free energetics resulting higher stability rather than other two amino acids i.e. glycine and D,L-alanine.This type of results might be due to the size factor of the amino acid and aqueous electrolyte mixtures. The dipole moment and size of D,L-nor-valine (6.92 Å) [27, 34] matched properly to interact like dipole-dipole or to create cavity with the aqueous K_2_SO_4_ molecules (5.92 Å) [19] respectively. On the other hand, DL-alanine gets third highest stability in terms of cavity forming interaction in whole range of electrolyte concentration due its size factor but the dipole-dipole interaction does not supports its more stability in higher concentration of electrolyte. Resulting more stability of glycine in higher content of K_2_SO_4_ molecules in water due moment due to involvement of chemicals interactions i.e. the overall stability of D,L-alanine becomes fourth in position among the present amino acids in aqueous K_2_SO_4_solutions. The amino acid glycine shows slight higher stability in higher content of K_2_SO_4_ moleculesin solution due to chemical types of interactions.

## 4 Conclusion

The present study showed that of solubility of the experimental amino acids in aqueous K_2_SO_4_ solution is as follows: glycine > D,L-alanine > D,L-nor-valine > D,L-serine. But the same trend of salting-in effect is as follows: D,L-alanine < glycine < D,L-nor-valine < D,L-serine. The fact is supported by salting-in and salting-out constants which are fully agreed with the actual chemical stability of the amino acids as: D,L-serine > D,L- nor-valine > glycine > D,L-alanine.

The solubility trends for first three amino acids in current study i.e. glycine > D,L-alanine > D,L-nor-valine have good agreement with our previous work with Na_2_SO_4_ [11], but reverse trends of transfer Gibbs free energetics were found. This interesting behavior might be due to different size factors of Na^+^ and K^+^ cations with common anion. From the above result it could be concluded that the chemical stability of the amino acids is governed by the size and nature of cation or anion of the solvent molecules and also directed by the suitable size of the amino acids and the hydrophobic or hydrophilic character of their side chain.

## Declarations

### Author contribution statement

Aslam Hossain: Analyzed and interpreted the data.

Kalachand Mahali: Performed the experiments.

Bijoy Krishna Dolui: Contributed reagents, materials, analysis tools or data.

Partha Sarathi Guin: Conceived and designed the experiments; Wrote the paper.

Sanjay Roy: Analyzed and interpreted the data; Wrote the paper.

### Funding statement

This research did not receive any specific grant from funding agencies in the public, commercial, or not-for-profit sectors.

### Competing interest statement

The authors declare no conflict of interest.

### Additional information

No additional information is available for this paper.
